# Sustainable P3HB:ZnO Composite Piezoelectric Nanofibers for AI‐Driven Gait Monitoring

**DOI:** 10.1002/smsc.70355

**Published:** 2026-07-29

**Authors:** Milad Razbin, Kexin Ruan, Danish Tahir, Xuan Li, Qing Li, Hala Zreiqat, Fariba Dehghani, Shuying Wu

**Affiliations:** ^1^ School of Aerospace Mechanical and Mechatronic Engineering The University of Sydney Sydney New South Wales Australia; ^2^ School of Biomedical Engineering The University of Sydney Sydney New South Wales Australia; ^3^ School of Chemical and Biomolecular Engineering The University of Sydney Sydney New South Wales Australia

**Keywords:** artificial neural network, electrospinning, genetic algorithm, piezoelectricity, sustainable materials

## Abstract

Degrable, high‐performance piezoelectric materials are critical for implantable and environmentally friendly devices capable of reliable sensing and energy harvesting while safely degrading after use. However, most degrable piezoelectric materials still suffer from limited electromechanical performance. Here, we report a high‐performance poly(3‐hydroxybutyrate):zinc oxide (P3HB:ZnO) composite piezoelectric nanofiber optimized using an integrated artificial neural network‐genetic algorithm framework. The model identified an optimal ZnO content of 4.3 wt.%, resulting in significantly enhanced piezoelectric performance compared with pristine P3HB. The optimized nanofibers achieved a peak voltage sensitivity of 0.382 mV/kPa at 1 Hz and an effective piezoelectric voltage coefficient of 13.65 mV/m·N, with stable output over 1000 loading cycles and a response delay of 318 ms. Biodegradation studies showed approximately 38% mass loss after 6 weeks, while the nanofibers retained measurable piezoelectric functionality within this timeframe. Furthermore, integration with a multichannel sensing platform enabled a proof‐of‐concept AI‐assisted gait‐recognition demonstration. This work presents a scalable machine‐learning‐driven strategy for designing high‐performance sustainable piezoelectric materials for self‐powered transient bioelectronics.

## Graphical abstract

This work presents an AI‐enabled framework for designing sustainable P3HB:ZnO piezoelectric nanofibers by integrating artificial neural networks and genetic algorithms to optimize material composition and electrospinning parameters. The developed nanofibers exhibit enhanced piezoelectric performance and are successfully integrated into an intelligent gait monitoring system, demonstrating their potential for sustainable sensing applications.

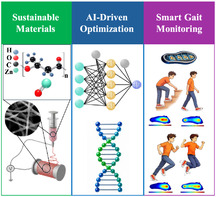

## 1 Introduction

Piezoelectric materials are essential for self‐powered sensing in wearable, biomedical, and human–machine interface systems owing to their ability to directly convert mechanical stimuli into electrical signals without the need for external power [1, 2]. Meanwhile, sustainable piezoelectric materials have emerged as a promising class of functional materials capable of safely degrading in both environmental and physiological conditions. Unlike conventional ceramic or polymer‐based piezoelectrics, which persist for prolonged periods and contribute to electronic waste, sustainable alternatives decompose into nontoxic byproducts, thereby eliminating end‐of‐life accumulation and mitigating ecological impact [3]. Their transient nature is particularly advantageous for sensors, stimulators, and diagnostic implants, as it enables operation over a defined period followed by safe resorption, thereby eliminating the need for secondary surgical removal and minimizing chronic inflammatory responses [4]. Moreover, some sustainable piezoelectric polymers can be tailored to possess tissue‐matched mechanical properties while maintaining effective electromechanical coupling, thereby enabling sensitive interfacing with biological forces [5]. These attributes position biodegradable piezoelectric materials as an ideal platform for sustainable energy harvesting, transient bioelectronic systems, and eco‐friendly wearable sensors.

Different biodegradable piezoelectric materials have been explored, including natural polymers such as cellulose [6], collagen [7], and chitosan [8]; synthetic biocompatible polyesters such as poly(3‐hydroxybutyrate) (P3HB) and its copolymers [9, 10] and poly(L‐lactic acid) (PLLA) [11, 12]. Both categories exhibit several important limitations. Natural piezoelectric biopolymers are intrinsically attractive due to their biocompatibility, sustainability, and structural similarity to native tissues. However, achieving consistent purity, structural control, and reproducible piezoelectric performance remains challenging. Synthetic biodegradable polymers can deliver stronger and more tunable piezoelectric responses, yet most are derived from petroleum‐based sources. Notably, P3HB stands out as promising future piezoelectric biodegradable polyester that can be biosynthesized through microbial fermentation, offering a renewable and scalable pathway while maintaining a well‐defined crystalline structure capable of strong dipolar alignment [3]. However, P3HB is inherently brittle, and its electromechanical performance remains relatively low and highly sensitive to processing conditions and crystalline phase [13]. To address these limitations, inorganic fillers are commonly incorporated to enhance piezoelectric performance through increased polarization [14, 15]. ZnO is distinguished as one of the few biocompatible and bioresorbable inorganic piezoelectrics, owing to its wurtzite crystal structure, high polarization capability, and intrinsic antimicrobial activity. These attributes, together with its safe degradation into physiologically acceptable Zn^2+^ ions, make ZnO an attractive reinforcing component for the development of fully biodegradable nanogenerators [16, 17].

Electrospinning has emerged as a powerful technique for fabricating piezoelectric materials, as it enables the production of ultrafine fibers with high surface area, controlled alignment, and tunable crystallinity, factors that critically influence electromechanical performance [18]. The intense stretching and rapid solidification inherent to electrospinning promotes molecular chain orientation and phase formation, allowing polymers such as P3HB and its composites to exhibit enhanced piezoelectric activity compared with bulk films [19]. Moreover, functional nanofiller incorporation has recently been shown to simultaneously enhance electromechanical performance and wearable sensing capability in electrospun polymer composites [20]. However, electrospinning is inherently multivariate and highly sensitive to solution, processing, environmental, and postprocessing parameters. Consequently, achieving optimal performance requires precise control over these variables, which is difficult to attain using conventional trial‐and‐error approaches [21, 22]. Artificial intelligence‐based optimization tools such as response surface methodology (RSM), artificial neural networks, and genetic algorithms provide a robust framework for addressing this challenge. Similarly, AI‐assisted signal processing has been integrated with wearable sensing platforms to improve physiological monitoring and disease recognition [23, 25].

**FIGURE 1:**
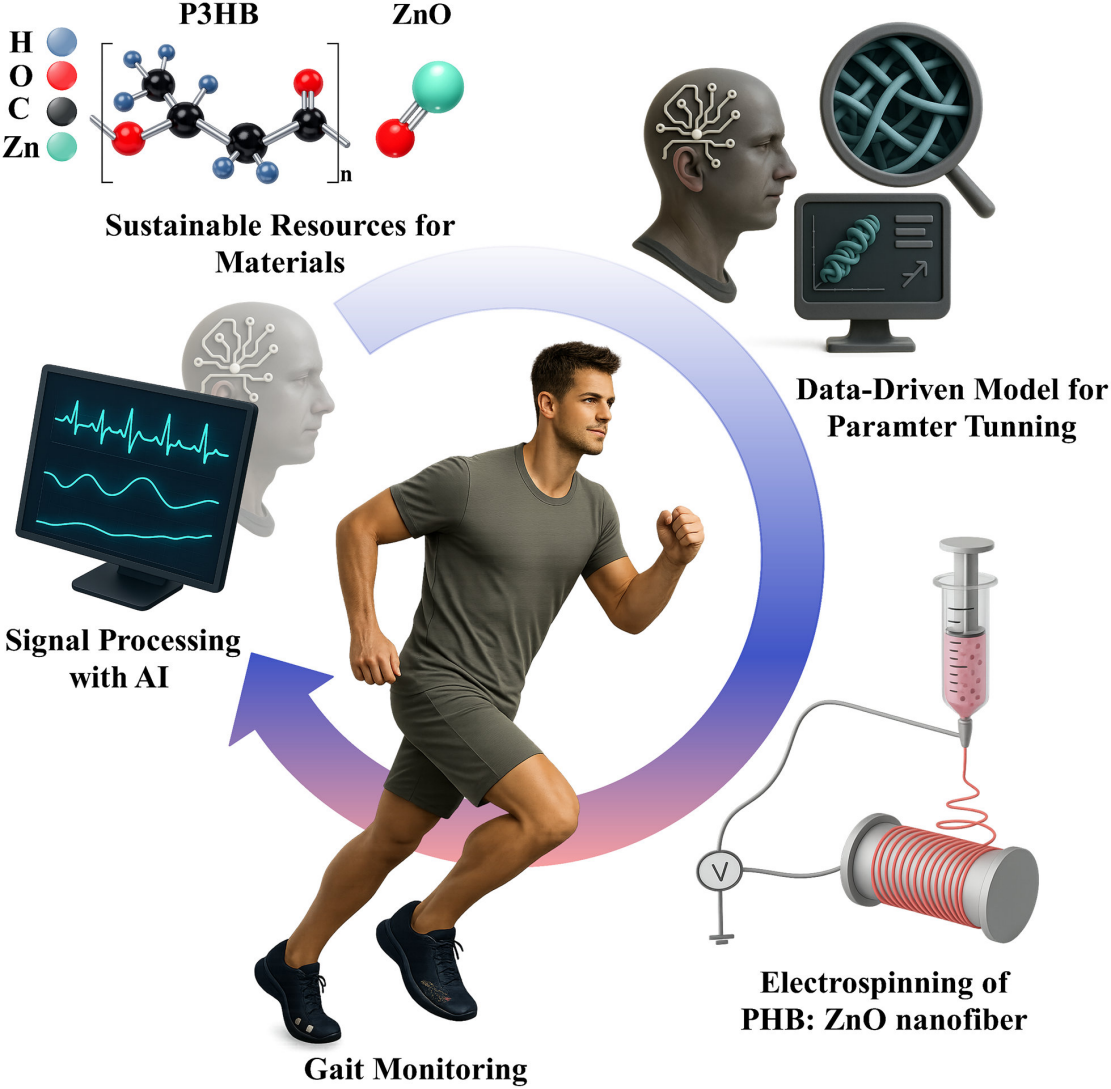
Conceptual illustration of the integrated framework and key outcomes of this study.

Herein, we present high‐performance piezoelectric P3HB:ZnO nanocomposites enabled by a data‐driven optimization strategy. Figure 1 outlines the complete workflow, including material selection, data‐driven optimization, electrospinning‐based nanofiber fabrication, and gait monitoring demonstrations. In this system, ZnO is incorporated as a functional reinforcement to simultaneously enhance polarization and mechanical robustness while maintaining sustainability. An artificial intelligence‐assisted framework integrates experimental data with predictive modeling to identify optimal electrospinning parameters and filler compositions, thereby maximizing electromechanical performance. This approach transforms electrospinning from an empirically guided process into a predictive and highly tunable manufacturing strategy for high‐performance piezoelectric nanofibers. The potential of the resulting P3HB:ZnO nanofiber mats is further demonstrated as a proof‐of–of‐concept wearable smart insole for AI‐assisted gait monitoring.

## 2 Experimental Section

### 2.1 Materials and Reagents

Poly(3‐hydroxybutyrate) (P3HB, Mw ∼80 kDa, <48 μm) was purchased from Echomann (China) Co., Ltd. Zinc oxide (ZnO, <50 nm) was ordered from Merck Life Science Co., Ltd (Australia). Ethanol (99.8%) and Chloroform (99.8%) were sourced from Thermo Fisher Scientific Co., Ltd (Australia).

### 2.2 Fabrication of P3HB: ZnO Composite Nanofiber

**FIGURE 2:**
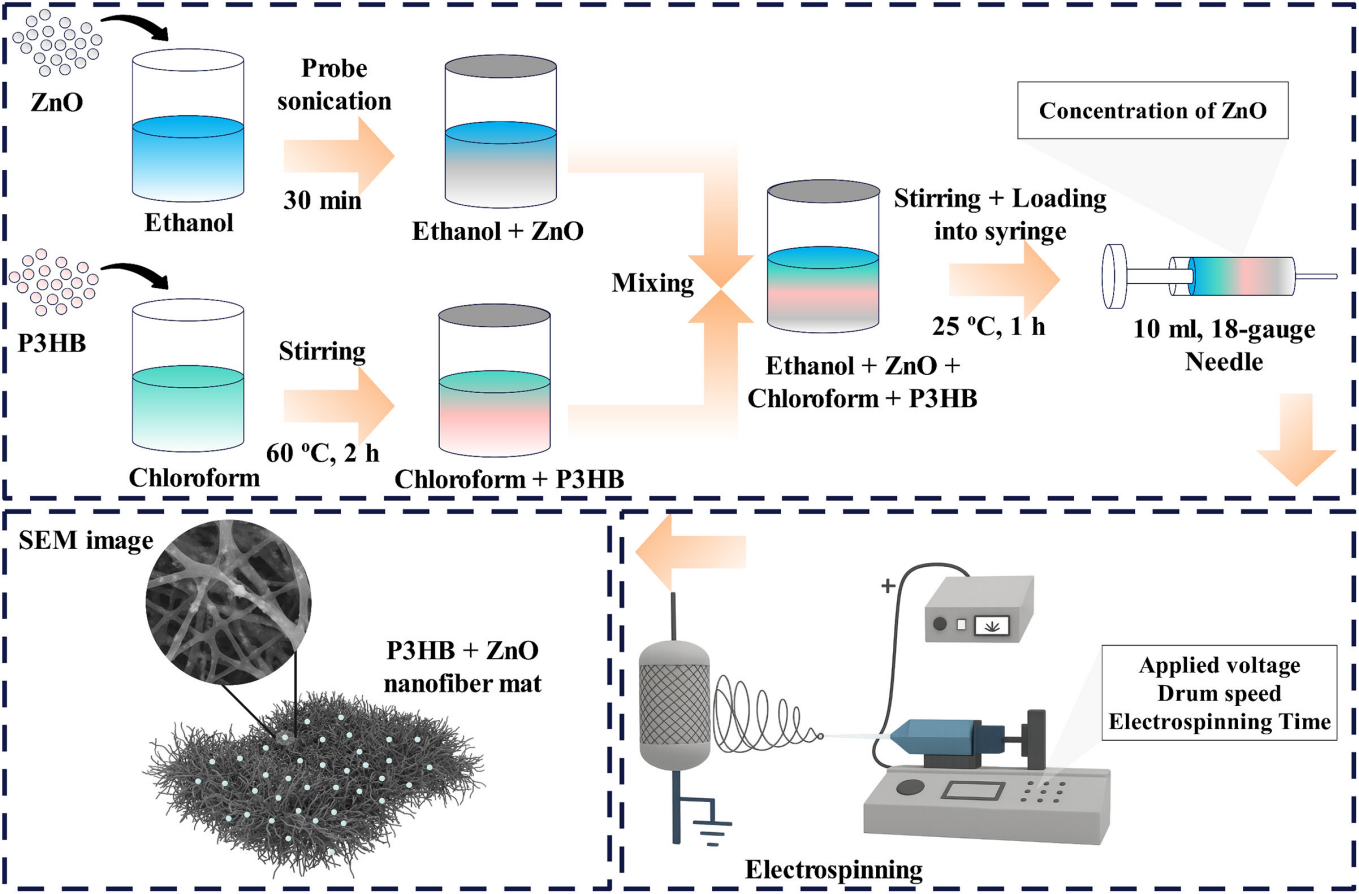
Schematic illustration of the fabrication process for the P3HB:ZnO composite piezoelectric nanofiber.

Figure 2 illustrates the workflow for solution preparation and electrospinning. To prepare the polymer solution, P3HB was first dissolved in chloroform under magnetic stirring at 60 °C for 2 h. In parallel, ZnO nanoparticles were dispersed in ethanol using probe sonication (40% power, Model CV334, Sonics Vibra‐Cell) for 30 min. The P3HB solution was subsequently added to the ZnO dispersion and stirred at 25 °C for 1 h to ensure homogeneity. The P3HB concentration was maintained at 10 wt.%, while the ZnO content was varied according to the experimental design. Nanofibers were fabricated using an electrospinning system (KATO TECH Co., Ltd.,18‐gauge needle) with a needle‐to‐collector distance of 10 cm, a feed rate of 1 mL/h, a transverse movement speed of 30 cm/min, a relative humidity of 60%, and an environmental temperature of 26 °C.

### 2.3 Characterization Methodologies

The open‐circuit voltage ($V_{\mathrm{OC}}$) under compressive stress ($\sigma_{c}$) was measured on 3 × 3 cm^2^ samples sandwiched between two aluminum foil electrodes and connected to a portable oscilloscope (Analog Discovery 2) integrated with a customized linear compression stage. Under the same loading conditions, the short‐circuit current ($I_{\mathrm{SC}}$) was recorded using a low‐noise current preamplifier (SR570, Stanford Research Systems) with variable resistance. To minimize triboelectric interference, a grounded conductive shielding layer was applied following previously reported protocols [26, 27]. Furthermore, the nanofiber mat was tested in a fixed sandwich‐electrode configuration under normal compressive loading, minimizing relative sliding between contact interfaces. Consequently, the recorded electrical output predominantly originated from the piezoelectric response of the P3HB:ZnO nanofibers. For consistent comparison of piezoelectric performance, $V_{\mathrm{OC}}$ was normalized by $\sigma_{c}$, yielding the normalized voltage ($V_{n}$), as defined in Equation (1).

(1) $$V_{n}\left(mv/kPa\right)=\;\frac{V_{\mathrm{OC}}}{\sigma_{c}}$$

Note that the stress used for these tests is approximately 150 ± 30 kPa. The microstructure of the electrospun nanofiber mats was characterized using scanning electron microscopy (SEM; Phenom XL G2, Thermo Fisher Scientific) operated at an accelerating voltage of 5 kV, with varying magnifications to capture both overall morphology and fine surface features. Attenuated total reflectance‐Fourier transform infrared, (ATR‐FTIR, Nicolet iS50, Thermo Fisher Scientific) spectra were acquired over a wavenumber range of 4000–600 cm^−1^ with 256 scans at a resolution of 4 cm^−1^ to ensure a high signal‐to‐noise ratio. X‐ray diffraction (XRD; Miniflex 600C, Rigaku) was performed using Cu Kα radiation (λ = 1.5406 Å) over a 2θ range of 5°‐80°, with a step size of 0.02° and a scanning rate of 2° min^−1^ to achieve sufficient peak resolution. Water contact angle (WCA; Theta Flex Auto 2, Biolin Scientific) measurements were conducted at 20 °C using the sessile drop method with an optical tensiometer.

### 2.4 Data Collection Methodology, Model Development and Optimizer

This study investigates the effectiveness of artificial intelligence in optimizing electrospinning parameters to enhance the piezoelectric performance of P3HB:ZnO composite piezoelectric nanofiber. A systematic experimental design is therefore essential to generate reliable datasets for model training and validation. Although multiple factors influence the electrospinning process, the primary determinants of the resulting piezoelectric response include ZnO content, nanofiber diameter, nanofiber orientation, and nanofiber mat thickness. To regulate these characteristics, four key processing parameters were selected: ZnO concentration, applied voltage, drum rotation speed, and electrospinning time. The operating ranges for these variables were established through preliminary experiments to ensure stable spinnability while enabling meaningful variation in material properties. The selected parameters and their corresponding ranges are summarized in Table 1.

**TABLE 1:** List of control parameters for the electrospinning process.

Parameter	Unit	Level				
ZnO concentration	*wt.%*	0	7.5	15	22.5	30
Applied voltage	*kV*	8	12	16	—	—
Drum speed	*m/min*	1	5	9	—	—
Electrospinning time	*h*	3.5	7	10.5	—	—

To reduce experimental time and cost while maintaining a representative sampling of the design space, RSM was employed to select 15 representative combinations from the full set. These experimental conditions are summarized in Table 2.

**TABLE 2:** RSM‐selected parameters and piezoelectric performance of P3HB:ZnO composite piezoelectric nanofiber.

Sample	ZnO Con., wt.%	Applied voltage, kV	Drum speed, m/min	Electrospinning time, h	Normalized Voltage, mV/kPa
1	30.0	8	1	3.5	0.046
2	30.0	16	5	7.0	0.094
3	15.0	12	5	3.5	0.054
4	30.0	12	1	10.5	0.075
5	0.0	8	5	3.5	0.155
6	7.5	12	9	7.0	0.131
7	22.5	8	5	7.0	0.097
8	30.0	12	9	10.5	0.068
9	15.0	16	5	10.5	0.192
10	0.0	12	5	10.5	0.135
11	0.0	16	9	3.5	0.069
12	0.0	16	1	7.0	0.116
13	30.0	8	9	3.5	0.097
14	7.5	8	1	10.5	0.078
15	7.5	8	9	10.5	0.144
Min	0	8	1	3.5	0.046
Max	30	16	9	10.5	0.192

In addition, ANN was employed as a more robust modeling approach due to its ability to capture complex, nonlinear relationships that are difficult to represent using conventional statistical models [28]. Bayesian optimizer (BO) was applied using RSM and ANN models as surrogates, enabling efficient exploration of the design space with reduced experimental cost [29].

To further enhance optimization performance, a GA was also employed. Based on evolutionary principles of selection and variation, GA is particularly effective in navigating complex, multimodal search spaces and can outperform BO in identifying global optima in challenging process systems [30]. The specific configurations and computational settings for each method are summarized in Table 3, including the RSM design (I‐optimal) and second‐order regression model implemented in Excel; the ANN architecture and training parameters defined in MATLAB; the BO acquisition function with a maximum of 200 iterations; and the GA parameters, including population size, selection, crossover, mutation, and migration settings. Initial parameter values were selected based on prior studies [31, 32], followed by further tuning to refine and finalize the optimal configurations. To further evaluate model reliability and reduce the influence of a single train‐test partition, the ANN model was additionally assessed using 7‐fold cross‐validation. The predictive performance obtained across all folds confirmed the consistency of the model within the investigated experimental design space.

**TABLE 3:** List of hyperparameter settings for various methods.

Method	Parameter	Value
RSM	Model	Second‐order polynomial regression
—	Type	I‐Optimal
—	Software used	Excel
ANN	Neural network	Feed‐forward back‐propagation
—	Number of neurons in hidden layer	5
—	Activation function of hidden layer	Tan‐sigmoid
—	Activation function of output layer	Pure linear
—	Learning rate	0.40
—	Momentum value	0.70
—	Learning function	Levenberg–Marquardt
—	Software used	MATLAB
BO	Acquisition function	Expected‐Improvement‐Plus (EI^+^)
—	Maximum evaluations	200 iterations
—	Software used	MATLAB
GA	Size of population	25
—	Type of population	Double vector
—	Generation number	100
—	Function of creation	Uniform
—	Function of scaling	Rank
—	Function of selection	Roulette
—	Elite number	1
—	Function of mutation	Adapt feasible
—	Fraction of mutation	0.10
—	Function of crossover	Two points
—	Fraction of crossover	0.40
—	Migration direction/interval	Forward/20% of population size
—	Fraction of migration	0.10
—	Software used	MATLAB

## 3 Results and Discussion

### 3.1 Performance and Pattern Recognition of Data‐Driven Models

**FIGURE 3:**
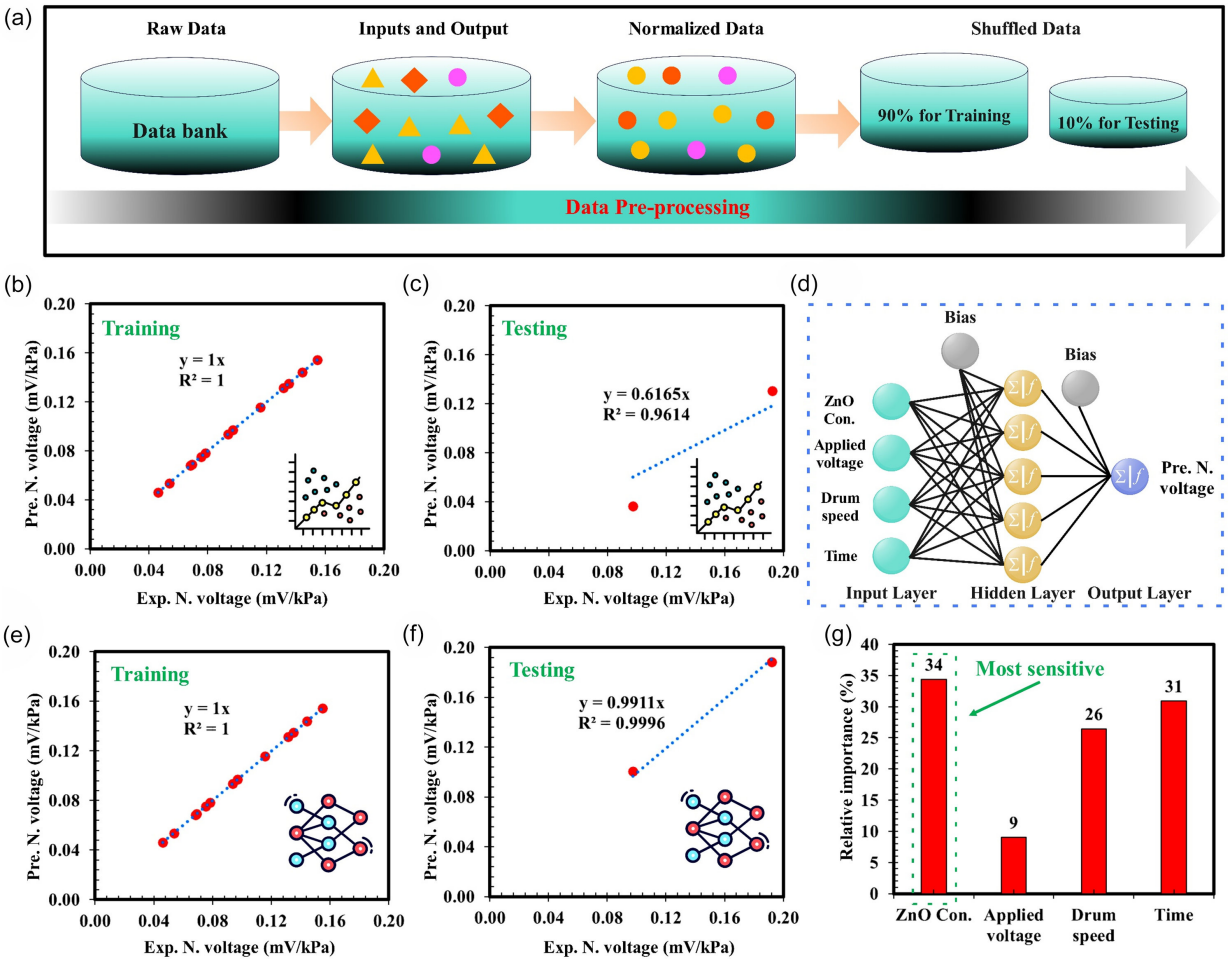
Data‐driven modeling and performance evaluation of the normalized voltage response: (a) schematic representation of the data preprocessing pipeline, including data banking, input–output selection, normalization, shuffling, and dataset partitioning into training (90%) and testing (10%) subsets; (b,c) training and testing performance of the RSM model, showing the correlation between experimentally measured and predicted normalized voltage values; (d) architecture of the ANN, illustrating the input parameters (ZnO concentration, applied voltage, drum speed, and electrospinning time), hidden layers with bias terms, and the output normalized voltage; (e,f) training and testing performance of the ANN model, demonstrating improved prediction accuracy compared with the RSM model; (g) sensitivity analysis of the ANN model, highlighting the relative importance of each processing parameter on the normalized voltage output.

Figure 3a illustrates the complete data preprocessing workflow, beginning with the acquisition of experimental data (Table 2), followed by normalization of all input and output variables to the range of 0.1–0.9 to ensure numerical stability during model training. The dataset was then randomly shuffled to eliminate ordering bias and preserve statistical independence and subsequently divided into training (90%) and testing (10%) subsets to evaluate model generalization. Figure 3b–c present the performance of the RSM model. Although the training fit appears ideal (R^2^ = 1), the testing results, characterized by a slope of 0.6165 and R^2^ = 0.9614, indicate limited predictive generalization and an inability to fully capture the inherent nonlinearity between electrospinning parameters and piezoelectric output. In contrast, the ANN architecture shown in Figure 3d, comprising four input neurons, a nonlinear hidden layer, and a linear output neuron, suggests superior predictive capability. As illustrated in Figure 3e–f, the ANN achieves near‐perfect prediction accuracy for both training and testing datasets (R^2^ ≈ 1 and slopes ≈ 1) for fold 1, confirming its effectiveness in modeling complex multiparameter interactions. The sensitivity analysis in Figure 3g quantifies the relative importance of each parameter [33], identifying ZnO concentration (34%) and electrospinning time (31%) as the dominant factors, followed by drum speed (26%) and applied voltage (9%). Given its superior predictive performance and ability to capture nonlinear interactions, the ANN model was selected as the primary predictive framework and subsequently coupled with optimizers for robust multiparameter optimization. The results of the 7‐fold cross‐validation further confirmed the robustness of the ANN model, with all validation folds achieving R^2^ values exceeding 0.99, demonstrating consistent predictive capability within the investigated parameter space.

**FIGURE 4:**
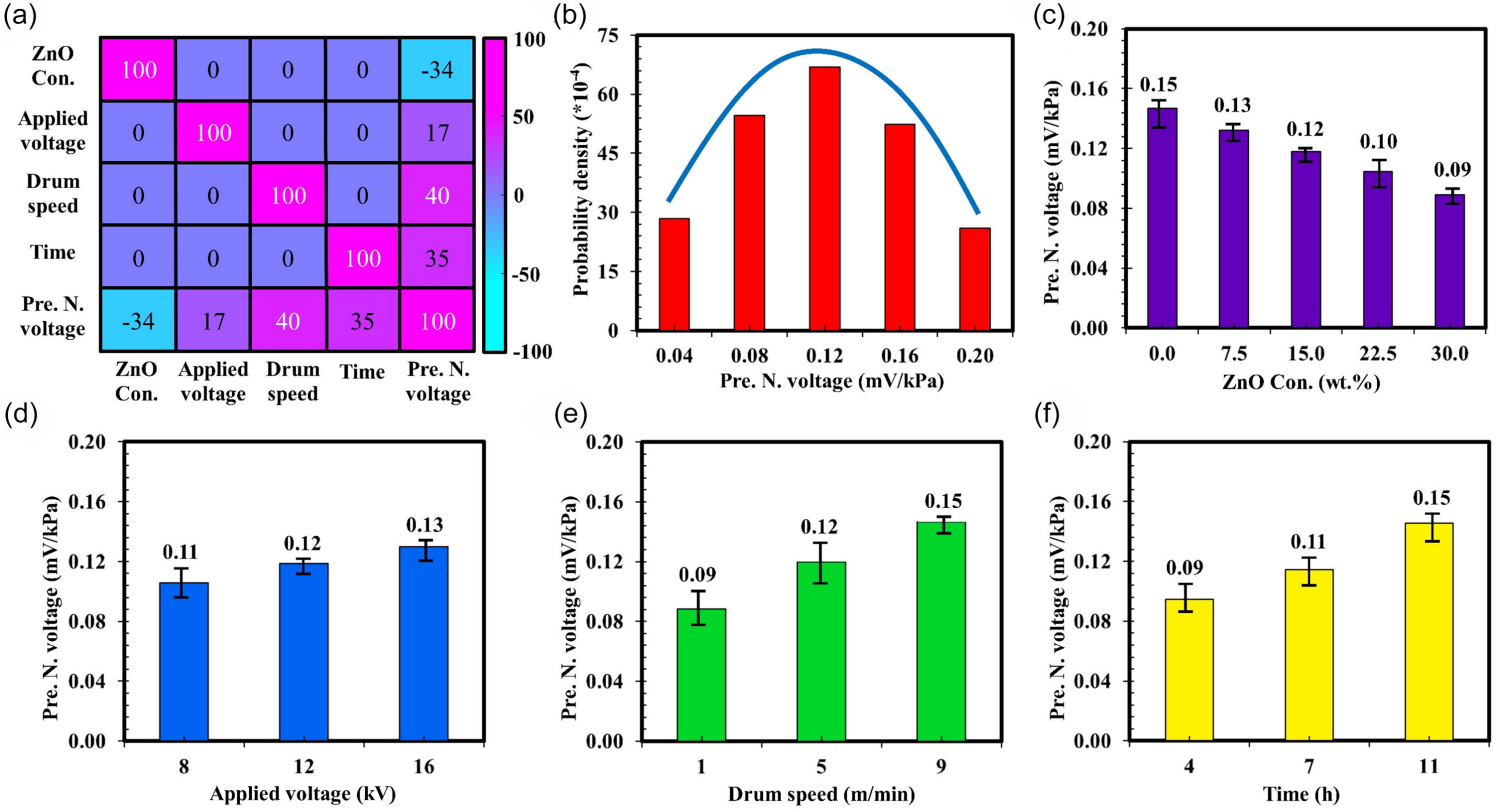
Statistical analysis of ANN‐GA‐predicted normalized voltage output: (a) Pearson’s correlation heatmap illustrating the relationships between ZnO concentration, applied voltage, drum speed, electrospinning time, and the predicted normalized voltage; (b) probability distribution of the predicted normalized voltage values obtained from the optimized model; parametric influence of individual processing variables on the predicted normalized voltage: (c) ZnO concentration, (d) applied voltage, (e) drum speed, and (f) electrospinning time.

The statistical characterization of the ANN‐generated dataset, obtained by evaluating all possible parameter combinations within the defined design space, reveals the underlying trends and response patterns learned by the model. As shown in Figure 4a, Pearson’s correlation heatmap derived from the complete predicted dataset highlights clear relationships between electrospinning parameters and the resulting normalized voltage output. A negative correlation is observed between normalized voltage and ZnO concentration (−34%, moderate negative correlation), whereas positive correlations are identified for applied voltage (17%, weak positive correlation), drum speed (40%, moderate positive correlation), and electrospinning time (35%, moderate positive correlation). These trends underscore the influence of ZnO content, nanofiber diameter and alignment, and mat thickness on piezoelectric performance [34]. The predicted probability distribution (Figure 4b) follows a near‐Gaussian profile centered at approximately 0.12 mV/kPa, indicating stable and consistent model predictions across the explored parameter space. Figure 4c–f further illustrate the predicted influence of individual processing parameters on normalized voltage. As shown in Figure 4c, the normalized voltage decreases monotonically with increasing ZnO concentration, from 0.15 to 0.09 mV/kPa within the experimentally investigated range. It should be noted that this trend reflects the discrete parameter combinations defined by the experimental design; intermediate concentrations were not explicitly evaluated. In composite piezoelectric systems, nanoparticle incorporation is typically governed by optimal concentration, at which a uniform dispersion of fillers forms and maximizes electromechanical response [35, 37]. Below this threshold, the filler content is insufficient to establish effective dipolar coupling, whereas above it, particle agglomeration, dielectric screening, and mechanical discontinuities can degrade performance. Therefore, the apparent monotonic decrease observed here does not preclude the existence of optimal ZnO concentration. Instead, it suggests that the percolation threshold lies within a narrower compositional window not fully resolved by the experimental sampling. To address this, the analysis was extended through model‐based optimization to identify the optimal filler concentration corresponding to the percolation regime.

In contrast, increasing the applied voltage (Figure 4d) results in a moderate enhancement in normalized voltage (0.11 to 0.13 mV/kPa), attributed to intensified electric‐field‐driven stretching of the polymer jet, which promotes molecular chain orientation and dipole alignment within the P3HB matrix [38]. Drum speed (Figure 4e) exhibits a pronounced positive effect, with normalized voltage increasing from 0.09 to 0.15 mV/kPa as rotational speed increases. Higher drum speeds improve nanofiber alignment along the collector, facilitating more efficient stress transfer and cooperative dipole orientation under mechanical loading, thereby enhancing piezoelectric response [34]. Similarly, increasing electrospinning time (Figure 4f) leads to a progressive increase in normalized voltage. Longer spinning durations yield thicker nanofiber mats, enabling greater collective charge generation during deformation and resulting in increased voltage output [34]. Overall, the ANN‐derived statistical analysis suggests the model’s ability to capture complex nonlinear interactions among ZnO concentration, electric field strength, fiber alignment, and mat thickness. By reconstructing the full response landscape of the electrospinning process, the ANN provides a reliable predictive framework for the investigated electrospinning parameter space for identifying optimal conditions and guiding the design of high‐performance biodegradable P3HB:ZnO composite piezoelectric nanofibers.

### 3.2 Parameter Tuning of Electrospinning

**FIGURE 5:**
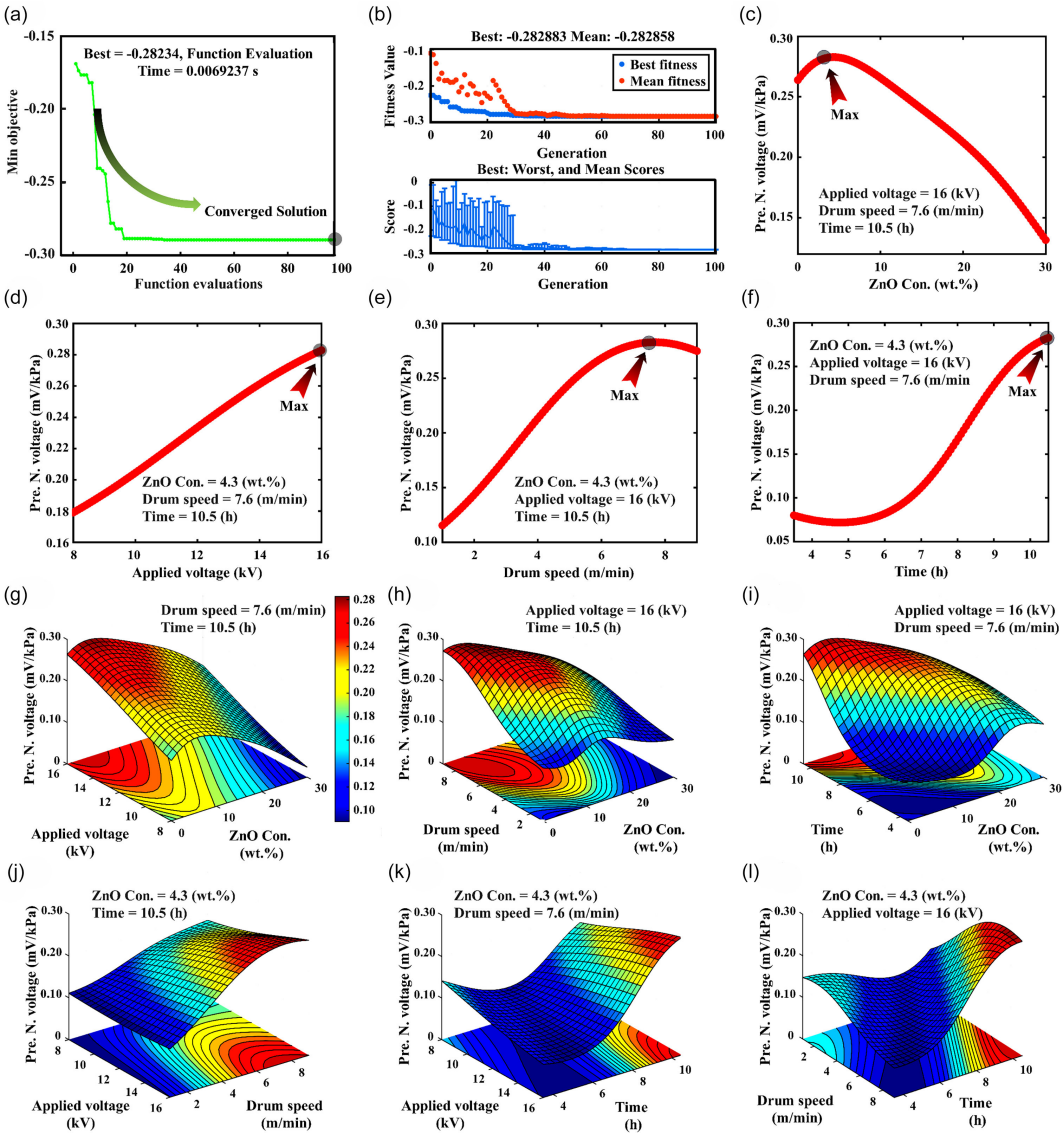
Optimization and response‐surface analysis of processing parameters governing the normalized voltage output of piezoelectric nanofibers: (a) convergence of the BO algorithm; (b) evolution of GA performance, with best, mean, and worst fitness values converging toward the global optimum; predicted single‐parameter response curves under optimized conditions, illustrating the effects of (c) ZnO concentration, (d) applied voltage, (e) drum speed, and (f) electrospinning time on normalized voltage, with arrows indicating optimal operating points; three‐dimensional response surface plots showing pairwise interactions between processing parameters and their combined influence on normalized voltage: (g) applied voltage versus ZnO concentration, (h) drum speed versus ZnO concentration, (i) electrospinning time versus ZnO concentration, (j) applied voltage versus drum speed, (k) applied voltage versus electrospinning time, and (l) drum speed versus electrospinning time. Color contours indicate the magnitude of the predicted normalized voltage response.

FIgure 5a–b illustrate the convergence behavior of BO and the GA, respectively. BO rapidly approaches a near‐optimal region within the first 20 function evaluations, reducing the objective value to approximately 0.28234 before gradually stabilizing. In contrast, GA exhibits a broader and more exploratory search trajectory over 100 generations, achieving a superior best fitness value of −0.282883 while maintaining a consistent decline in both mean and worst fitness values.

This behavior reflects the enhanced robustness of GA in navigating complex, multimodal landscapes and avoiding premature convergence, thereby providing a more reliable strategy for global optimization of electrospinning parameters. Figure 5c–f present one‐dimensional response curves derived from ANN‐predicted outputs, with all other parameters fixed at their optimized values. The model predicts a pronounced nonlinear decrease in normalized voltage with increasing ZnO concentration, from 0.28 mV/kPa at the optimal 4.3 wt.% to 0.09 mV/kPa at 30 wt.%, corresponding to a 68% reduction. This decline is attributed to excessive nanoparticle loading, which disrupts polymer matrix continuity, increases charge‐scattering interfaces, and hinders the formation of effective dipole pathways [39]. In contrast, applied voltage exhibits a monotonic positive effect, increasing the normalized voltage from 0.22 mV/kPa to 0.30 mV/kPa over the 8–16 kV range, consistent with enhanced electric‐field‐induced jet stretching and molecular orientation [40]. Drum speed shows one of the most significant influences, with normalized voltage increasing by over 60% between 1 and 9 m/min, reflecting improved fiber alignment and charge distribution. Similarly, electrospinning time has a strong positive effect, with normalized voltage increasing from 0.20 at 4 h to 0.31 mV/kPa at 10.5 h, indicating that thicker nanofiber mats facilitate more effective charge accumulation.

Figure 5g–l extend the analysis to two‐parameter interactions through three‐dimensional response surfaces. These plots reveal pronounced nonlinear curvature and strong interaction effects. For instance, ZnO concentration exhibits a saddle–shaped interaction with both applied voltage and drum speed, where performance peaks at low‐to‐moderate concentrations but deteriorates rapidly at higher loadings, irrespective of operating conditions. Similarly, the applied voltage and electrospinning time surface reveals an interaction region in which simultaneous increases in both parameters enhance performance more strongly than their individual trends, indicating a nonlinear, nonadditive coupling not captured by one‐dimensional analysis. The drum speed‐electrospinning time interaction map displays a ridge‐like region, where concurrent increases yield the highest predicted voltages, confirming their cooperative roles. Importantly, these interaction patterns are entirely reconstructed from ANN‐generated predictions, enabling interpretability of the model beyond conventional black‐box behavior. Coupling this predictive capability with GA optimization identifies a global optimum at 4.3 wt.% ZnO, 16 kV applied voltage, 7.6 m/min drum speed, and 10.5 h electrospinning time, yielding a maximum predicted normalized voltage of 0.2829 mV/kPa.

To experimentally validate the predictive capability of the ANN‐GA framework, a targeted comparison was conducted using three representative samples. Table 4 summarizes the processing parameters together with the corresponding predicted and experimental normalized voltage outputs. The experimentally guided sample was fabricated using 15 wt.% ZnO, an applied voltage of 16 kV, a drum speed of 5 m/min, and an electrospinning time of 10.5 h. This condition corresponds to an existing sample within the original dataset and represents the best‐performing configuration identified through experimental exploration. The ANN‐GA‐guided sample was fabricated based on the model‐predicted optimal parameters: 4.3 wt.% ZnO, 16 kV applied voltage, 7.7 m/min drum speed, and 10.5 h electrospinning time. The model predicted a normalized voltage of 0.283 mV/kPa, while the experimentally measured value reached 0.306 mV/kPa, corresponding to a prediction error of 8.13%. Finally, a reference sample containing no ZnO (0 wt.%) was prepared under the same optimized operational conditions (16 kV, 7.7 m/min, 10.5 h) to isolate the contribution of ZnO incorporation. This three‐way comparison enables direct evaluation of the model’s predictive accuracy and the physical validity of the optimized parameters. The close agreement between predicted and experimental outputs for the ANN‐GA‐guided sample confirms the robustness of the model, while benchmarking against the reference sample highlights the effectiveness of ZnO incorporation, resulting in an approximately 201% improvement in the piezoelectricity of P3HB.

**TABLE 4:** Comparison of control parameters for the three main samples.

Sample	ZnO, wt.%	Voltage, kV	Drum speed, m/min	Time, h	Pre. N. voltage, mV/kPa	Exp. N. voltage, mV/kPa	Error, %
ZnO‐Free	0	16	7.7	10.5	—	0.152	—
ANN‐GA	4.3	16	7.7	10.5	0.283	0.306	8.13
Exp.	15	16	5	10.5	0.192	0.192	0.0

### 3.3 Comprehensive Analysis of P3HB: ZnO Composite Nanofibers

#### 3.3.1 Mechanical, Physical Property, and Structural Analysis

**FIGURE 6:**
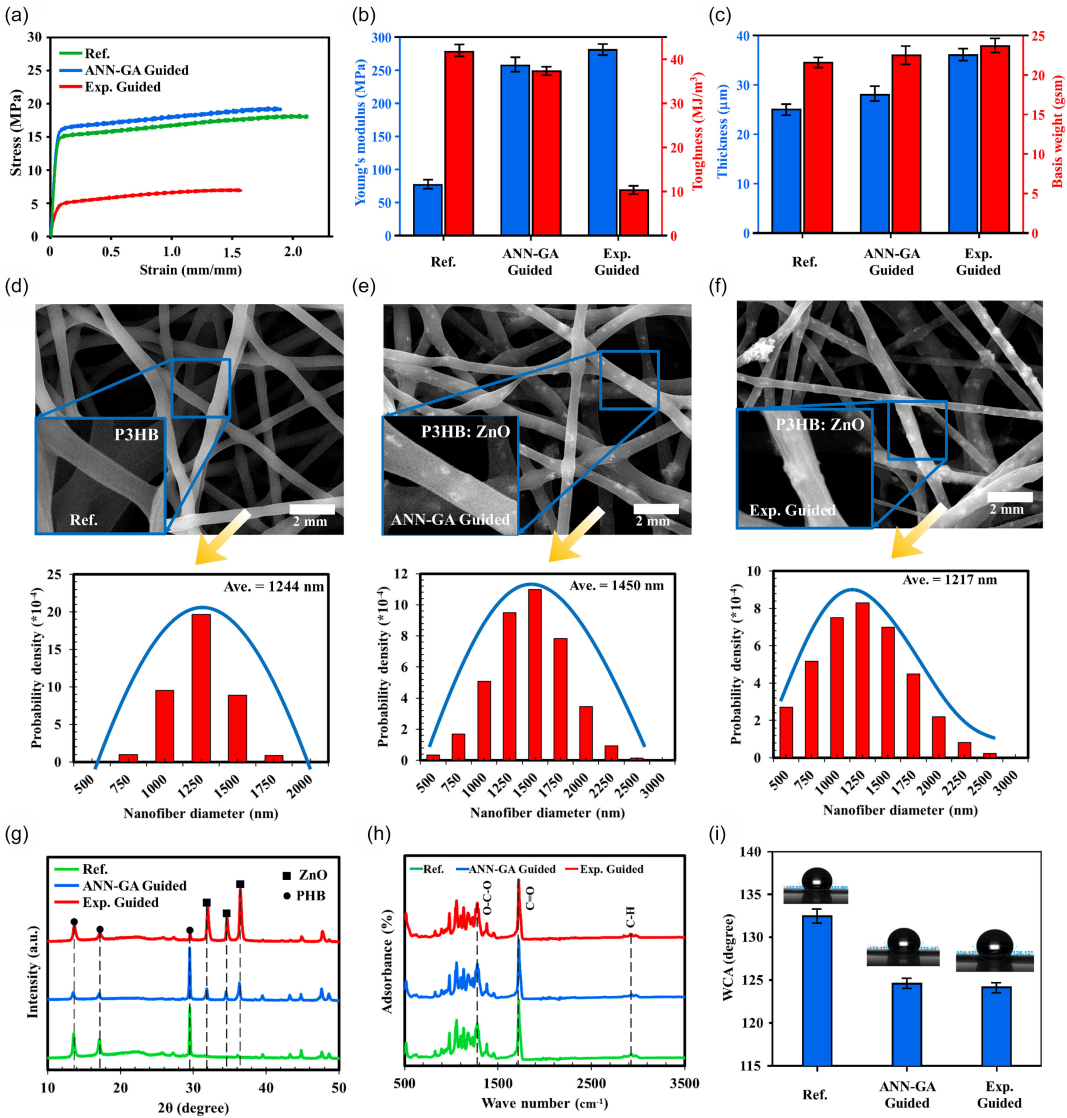
Comparison of the mechanical, physical, structural, and surface properties of the nanofibrous mats: (a) stress–strain curves of the three samples; (b) comparison of Young’s modulus and toughness (indicated by the area under the stress–strain curves); (c) thickness and basis weight; representative SEM micrographs and corresponding nanofiber diameter distributions of: (d) reference P3HB mats, (e) ANN‐GA‐guided P3HB:ZnO, and (f) experimental‐guided P3HB:ZnO; (g) XRD, (h) FTIR spectra, and (i) static water contact angle of the nanofiber mats.

Stress–strain profiles in Figure 6a reveal distinct mechanical responses across the three samples. The ANN‐GA‐guided sample suggests the highest tensile strength at break, indicative of a mechanically robust nanofiber mat. This behavior can be attributed to the optimized ZnO content (4.3 wt.%) and improved ZnO nanoparticle dispersion in PHB fibers. In contrast, the experimental‐guided sample with higher ZnO loading (15 wt.%) exhibits lower tensile strength and failure strain. In addition, the reference, pristine P3HB sample, shows lower tensile strength compared to the P3HB containing 4.3 wt.% ZnO but exhibits the highest stretchability, reflecting the intrinsic ductile nature of the nanofiber mat in the absence of rigid fillers [41]. As summarized in Figure 6b, the ANN‐GA‐guided sample achieves an intermediate modulus coupled with high toughness, confirming that the optimized parameter set effectively balances reinforcement and ductility, thereby enhancing the overall mechanical robustness of the nanofiber mat.

Using the relationship $\alpha =\frac{W}{t\rho_{f}}$ [42], the solidity (α) values were calculated from the measured basis weight (W), fiber density ($\rho_{f}$), and nanofiber mat thickness (t) (Figure 6c). The sample with 15 wt. % ZnO exhibits the lowest solidity ($\alpha_{\mathrm{Exp}.}$= 0.345), indicating a highly porous and loosely packed mat, which is consistent with the presence of agglomerated ZnO at 15 wt.% that disrupts uniform fiber packing. The reference sample shows the highest solidity ($\alpha_{\mathrm{Ref}.}$ = 0.690), reflecting a more compact and densely arranged nanofiber network in the absence of ZnO. The ANN‐GA‐guided sample displays an intermediate solidity ($\alpha_{\mathrm{ANN}-\mathrm{GA}}$ = 0.559) with moderate porosity. This moderate solidity, combined with uniform morphology and well‐dispersed ZnO, supports the enhanced toughness observed in Figure 6a–b. SEM images in Figure 6d–f highlight the microstructural differences. The ANN‐GA‐guided sample exhibits the most uniform fiber morphology, with an average diameter of 1450 nm, indicating improved jet stability and fiber formation. The sample with 15 wt.% ZnO shows a lower average diameter (1217 nm) but with left‐skewed distribution. Meanwhile, the reference sample displays relatively uniform fibers with an average diameter of 1244 nm.

According to Figure 6g, all three samples exhibit the characteristic diffraction pattern of semicrystalline P3HB, with intense reflections at 2θ ≈ 13.6°, 17.1°, 20.3–20.5°, 21.7°, 25.7°, 27.3–27.5°, and 29.4–30.0°, which can be indexed to the (020), (110), (021), (101), (121), (040), and (200) planes of the orthorhombic P3HB unit cell. These results confirm that the crystalline structure of P3HB is retained after electrospinning and after ZnO incorporation. In the reference sample, only P3HB reflections are observed, whereas the P3HB:ZnO composite piezoelectric nanofiber samples display additional peaks at 2θ ≈ 31.8°, 34.4°, and 36.3°, corresponding to the (100), (002), and (101) planes of hexagonal wurtzite ZnO, indicating that the filler preserves its crystalline integrity within the fibrous mats [43, 45]. The relative intensity of these ZnO reflections increases with increasing ZnO loading. Overall, the XRD results demonstrate that both the P3HB and ZnO lattices are retained in the composites.

According to Figure 6h, the FTIR spectra of the three samples retain all characteristic bands of P3HB, including the strong ester C═O stretching near 1720 cm^−1^, the CH_3_ asymmetric bending at ∼ 1457 cm^−1^, and the C–O–C/C–C–O skeletal vibrations in the 1280–1090 cm^−1^ region [46, 47]. FTIR analysis suggests that ZnO incorporation does not chemically modify P3HB.

The WCAs shown in Figure 6i reveal distinct differences in surface wettability among the nanofibrous mats with and without ZnO. The reference P3HB sample exhibits the highest contact angle, approximately 132°, confirming its strongly hydrophobic character. This behavior is typical for semicrystalline P3HB due to its compact chain packing and limited surface‐exposed polar functionalities [48]. In comparison, the P3HB:ZnO composite piezoelectric nanofiber (ANN‐GA‐guided and the experimental‐guided samples) display lower and similar contact angles of approximately 125° and 124°, respectively, indicating enhanced surface wettability. The incorporation of ZnO nanoparticles, which possess higher intrinsic surface energy than the P3HB matrix, can locally enrich the fiber surface with polar sites, thereby increasing water affinity.

#### 3.3.2 Piezoelectric Performance

**FIGURE 7:**
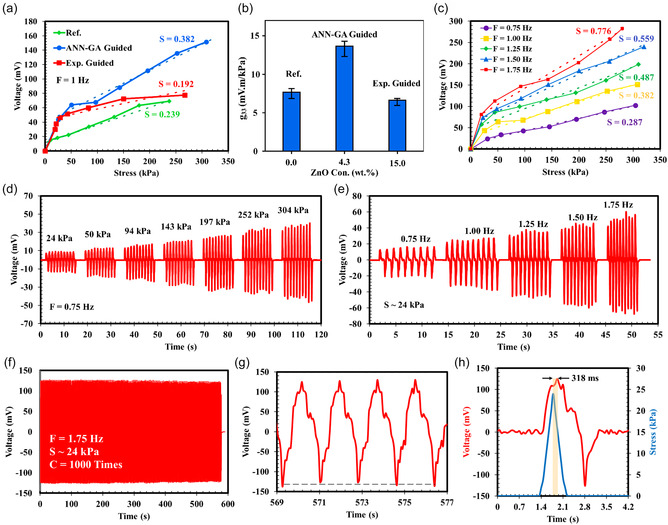
Piezoelectric performance of the nanofiber mats: (a) piezoelectric voltage output under different stresses applied at 1 Hz for the Experimental‐Guided, ANN‐GA‐Guided, and Reference samples; (b) comparison of the effective piezoelectric voltage coefficient as a function of ZnO concentration at 1 Hz; voltage‐stress response of the ANN‐GA‐guided sample (c) at different frequencies, (d) under stepwise increasing stress levels at a fixed frequency (0.75 Hz), (e) under stress of 24 kPa applied at different frequencies, (f) under stress of 24 kPa applied at 1.75 Hz over 1000 loading cycles, (g) enlarged view of the voltage signal at the final 4 cycles in (f) and (h) delay time between the applied stress and generated voltage.

As shown in Figure 7a, the output voltage increases linearly with applied stress for all samples with significantly different sensitivity. The ANN‐GA‐guided sample exhibits the highest slope (0.382 mV/kPa), which is nearly twice that of the experimental‐guided sample (0.192 mV/kPa) and approximately 60% higher than that of the reference mat (0.239 mV/kPa). The influence of ZnO concentration on the effective piezoelectric voltage coefficient (g_33_ = V_33_/(σ_33_t)), where V_33_ is the generated voltage along the poling (3) direction, *σ*_33_ is the applied mechanical stress in the same direction, and *t* is the thickness of the piezoelectric material, is quantified at frequency of 1 Hz in Figure 7b. The reference sample (0 wt.% ZnO) exhibits the lowest g_33_ value of 6.64 mV.m/N, consistent with the intrinsically weak piezoelectric response of P3HB. Incorporation of 4.3 wt. % ZnO (ANN‐GA‐predicted optimum) yields the highest g_33_ value of 13.65 mV.m/N, indicating that, at this composition, ZnO nanoparticles establish effective polarization pathways that maximize interfacial charge separation. In contrast, increasing the ZnO content to 15 wt. % (experimental‐guided) reduces g_33_ to 7.68 mV.m/N, suggesting that excessive filler loading disrupts uniform dispersion and promotes nanoparticle aggregation, thereby suppressing dipole mobility and weakening effective piezoelectric coupling.

Figure 7c presents the voltage‐stress response of the ANN‐GA‐guided sample over frequencies ranging from 0.75 to 1.75 Hz. Although linearity is preserved across all frequencies, the sensitivity increases markedly, from 0.287 mV/kPa at 0.75 Hz to 0.776 mV/kPa at 1.75 Hz. This frequency‐dependent enhancement is attributed to accelerated mechanical cycling, which enhances polarization dynamics while reducing charge dissipation between successive loading cycles [49]. Figure 7d–e show strong stress‐dependent signal scaling under stepwise loading from 24 to 304 kPa at 0.75 Hz. The bipolar voltage pulses exhibit a consistent shape with minimal hysteresis, demonstrating excellent mechanical reversibility. The voltage polarity was also consistently reversed during loading and unloading, which is characteristic of piezoelectric charge generation under compressive deformation. Similarly, Figure 7e shows the frequency‐dependent output under constant stress (24 kPa), where the signal amplitude increases from 0.75 to 1.75 Hz, consistent with the trend observed in Figure 7c. Long‐term cyclic stability is evaluated in Figure 7f, which presents the piezoelectric voltage output under a constant stress of 24 kPa applied at 1.75 Hz over 1000 cycles. The sample maintains a stable output of approximately ± 124.5 mV without observable degradation, confirming robust, stable piezoelectric behavior. The final four cycles (Figure 7g) remain nearly identical in peak amplitude and waveform, demonstrating excellent reproducibility. Figure 7h illustrates the temporal response of the piezoelectric signal under a single loading‐unloading cycle with the stress of 24 kPa applied at 1 Hz. The generated voltage closely follows the applied stress profile, with a measured response delay of 318 ms. This short delay indicates rapid electromechanical coupling and efficient charge generation in the nanofiber mat, confirming its suitability for dynamic sensing applications.

**FIGURE 8:**
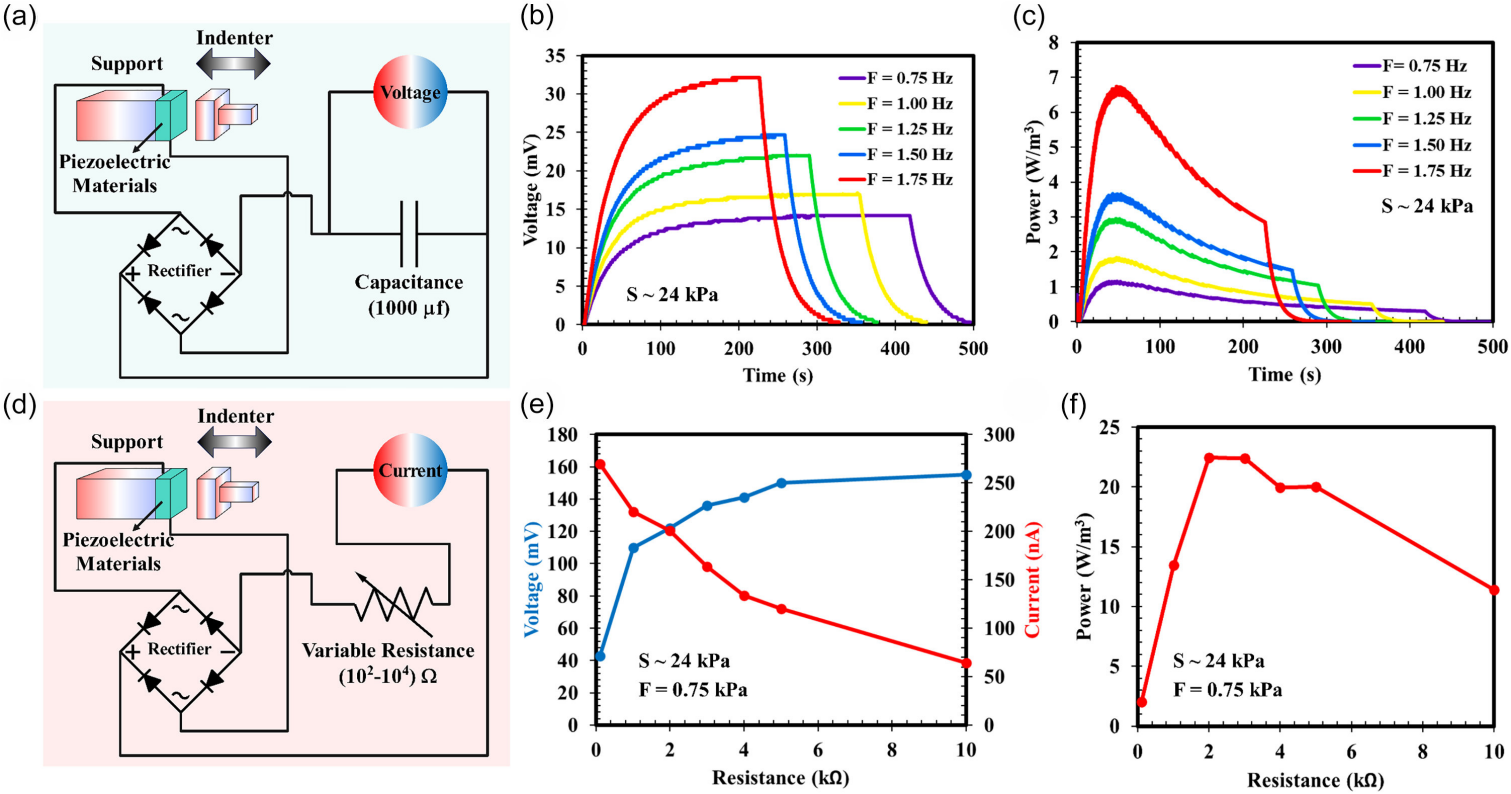
Energy‐harvesting performance of the ANN‐GA‐guided piezoelectric nanofiber mat: (a) schematic of the experimental setup for capacitor charging‐discharging measurements; (b) time‐dependent voltage evolution during capacitor charging under a stress of 24 kPa applied at varying frequencies and (c) the corresponding stored electrical energy in the capacitor. (d) Schematic of the setup for evaluating power delivery across an external variable load resistance; (e) output voltage and current, and (f) corresponding power density as a function of load resistance under a stress of 24 kPa applied at 0.75 Hz.

Figure 8a illustrates the capacitor‐charging configuration used to evaluate the energy‐harvesting capability of the piezoelectric nanofiber mats. A constant stress of 24 kPa was applied at different excitation frequencies, and the generated piezoelectric energy during cyclic loading‐unloading was stored in a capacitor (1000 µF). As shown in Figure 8b, the rectified output voltage increases systematically with excitation frequency, rising from 14.2 mV at 0.75 Hz to 31.78 mV at 1.75 Hz under constant stress. This frequency‐dependent enhancement reflects the increased rate of mechanical‐to‐electrical energy conversion, consistent with the dynamic piezoelectric response observed previously. The corresponding stored power density (Figure 8c) follows a similar trend, with peak values approaching 6.69 mW/m at 1.75 Hz, demonstrating the effective conversion of mechanical energy into usable electrical energy by the nanofiber mat. Figure 8d presents the experimental configuration used to evaluate power generation across external load resistances ranging from 100 to 100 kΩ for a nanofiber mat with a dimension of 2 × 2 cm^2^. As shown in Figure 8e–f, the output voltage increases monotonically with resistance, from 43 mV at 0.5 kΩ to 155 mV at 100 kΩ, while the current decreases sharply from 270 to 64 nA, reflecting the typical trade‐off between voltage and current in piezoelectric systems. The calculated power density reaches a maximum value of 22.45 mW/m at an optimal load resistance of 2 kΩ, indicating effective impedance matching between the device and external circuit.

As summarized in Table 5, the P3HB:ZnO composite piezoelectric nanofiber developed in this work exhibits a high voltage sensitivity of 0.776 mV/kPa at 1.75 Hz, outperforming previously reported ZnO‐reinforced polymer nanofibers.

**TABLE 5:** Comparison of electrospun piezoelectric nanofibers incorporating ZnO within polymeric matrices.

Materials	Max. sensitivity, mV/kPa	Frequency range, Hz	Pressure range, kPa	Ref.
PLLA:ZnO	0.110	3 Hz	0–34	[50]
PVDF:ZnO	0.370	4 Hz	150–750	[51]
P3HB:ZnO	0.776	0.75–1.75 Hz	0–305	This work

For comparison, PLLA:ZnO [50] and PVDF:ZnO [51] systems reported sensitivities of 0.11 and 0.37 mV/kPa, respectively, despite being evaluated at higher operating frequencies, where enhanced piezoelectric responses are typically expected. Notably, the P3HB:ZnO system achieves piezoelectric output nearly twice that of PVDF:ZnO while operating within a lower frequency range (0.75–1.75 Hz).

#### 3.3.3 Biodegradation Analysis

**FIGURE 9:**
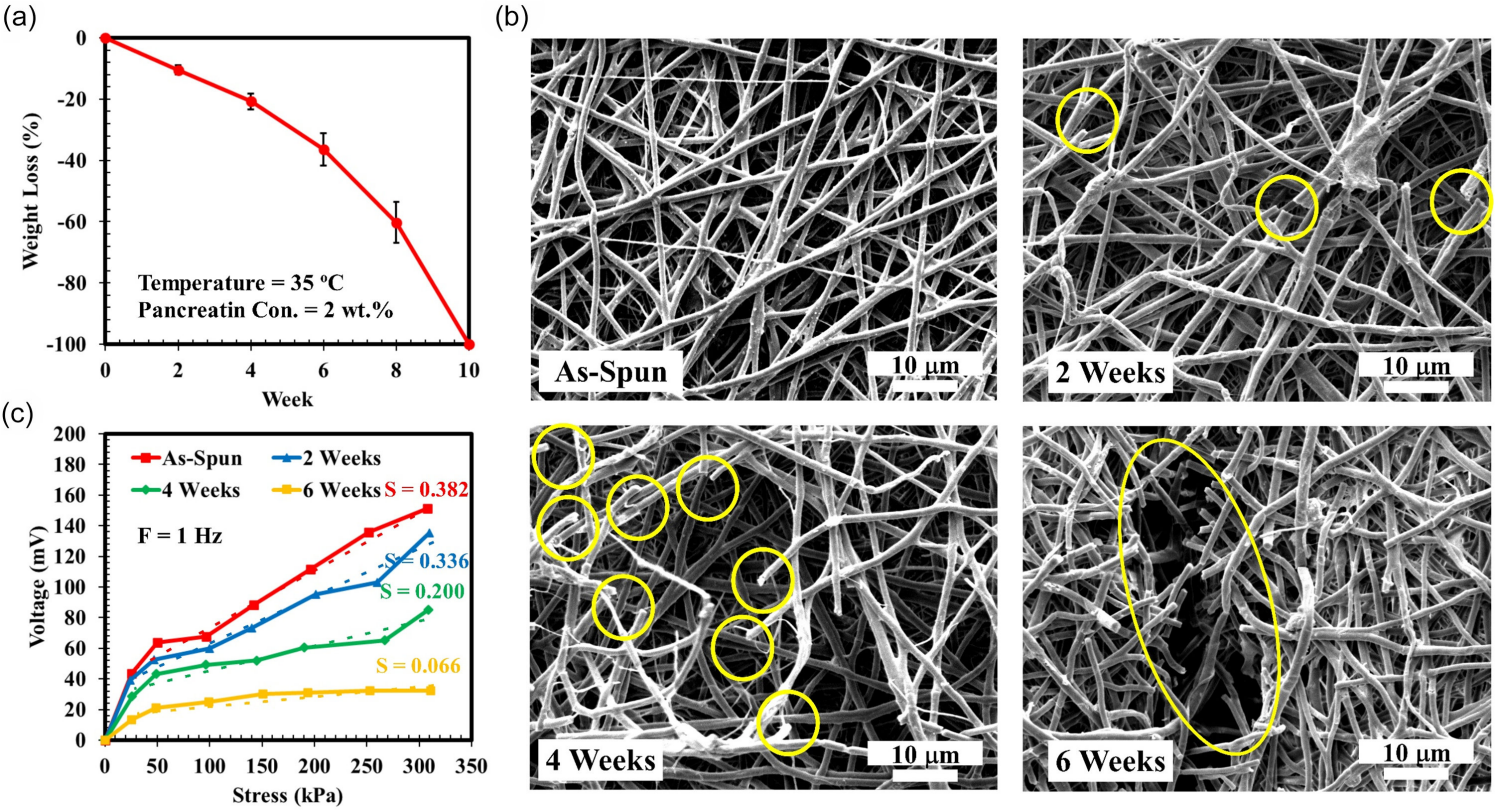
Degradation behavior of electrospun P3HB:ZnO composite piezoelectric nanofiber in PBS containing 2 wt.% pancreatin solution at 35 °C: (a) weight loss (%) of the nanofiber mats as a function of degradation time, (b) representative SEM micrographs of samples immersed in the solution for 0 day, 2 weeks, 4 weeks, and 6 weeks, and (c) evolution of the piezoelectric voltage output as a function of applied compressive stress applied at F = 1 Hz. Note that yellow markings in the SEM images indicate regions of localized damage and structural discontinuities.

The biodegradation study was conducted as a proof‐of‐concept evaluation to demonstrate the transient nature of the optimized P3HB:ZnO nanofibers under accelerated degradation conditions, that is, simulated enzymatically accelerated conditions in phosphate‐buffered saline (PBS) containing 2 wt.% pancreatin at 35 °C. Pancreatin, a mixture of proteases, lipases, and amylases, is known to accelerate the hydrolytic depolymerization of aliphatic polyesters. The weight‐loss profile (Figure 9a) confirms progressive degradation, with accelerated mass loss observed beyond 4 weeks and approaching near‐complete degradation by approximately 10 weeks. SEM micrographs (Figure 9b) reveal the corresponding microstructural evolution. After 2 weeks, mild localized structural degradation is observed, characterized by initial fiber breakage, indicating the onset of enzymatic attack. By week 4, degradation becomes more evident, with increased fiber fragmentation. After 6 weeks, extensive fiber breakage and cavity formation lead to pronounced structural disintegration, and by week 8–10 the nanofiber mat disintegrates into pieces and loses structural integrity. These morphological changes are attributed to enzymatic cleavage of ester bonds within the P3HB backbone, initiating surface erosion followed by bulk degradation as enzyme penetration increases. This behavior is consistent with previously reported degradation mechanisms of P3HB in enzyme‐rich environments [52]. During degradation, P3HB chains are progressively cleaved into shorter oligomers and ultimately into 3‐hydroxybutyrate monomers, which are water‐soluble and readily metabolized by microorganisms. These degradation products subsequently enter natural biochemical cycles and are converted into CO_2_ and H_2_O through microbial respiration, confirming the environmental compatibility of the material system [52].

Importantly, the incorporation of ZnO nanoparticles does not inhibit the biodegradation process. The gradual structural breakdown observed within 8–10 weeks suggests that the composite retains the intrinsic biodegradability of P3HB while possibly preserving its functional performance during the early stages of degradation. Further analysis revealed (Figure 9c) that the piezoelectric performance of the P3HB:ZnO composite piezoelectric nanofiber progressively diminishes during degradation, with voltage sensitivity decreasing from 0.383 to 0.066 mV/kPa at 1 Hz by week 6. This reduction is likely attributed to structural disintegration, loss of fiber connectivity, and disruption of effective dipole alignment as enzymatic degradation progresses. This result highlights the transient functional behavior of the material, demonstrating that it maintains effective piezoelectric performance during early stages while undergoing controlled degradation, making it highly suitable for temporary, self‐powered transient bioelectronic applications. It should be noted that the P3HB:ZnO nanofiber is designed as a transient biodegradable sensing material. Moreover, it should be mentioned that P3HB’s breakdown requires the presence of both moisture and specific depolymerase‐secreting microorganisms. Under normal ambient conditions, it can retain its mechanical and structural integrity for years [53]. So, its sensing performance would be expected to remain stable too.

### 3.4 Application Demonstration of P3HB: ZnO Composite Piezoelectric Nanofiber in Gait Monitoring

**FIGURE 10:**
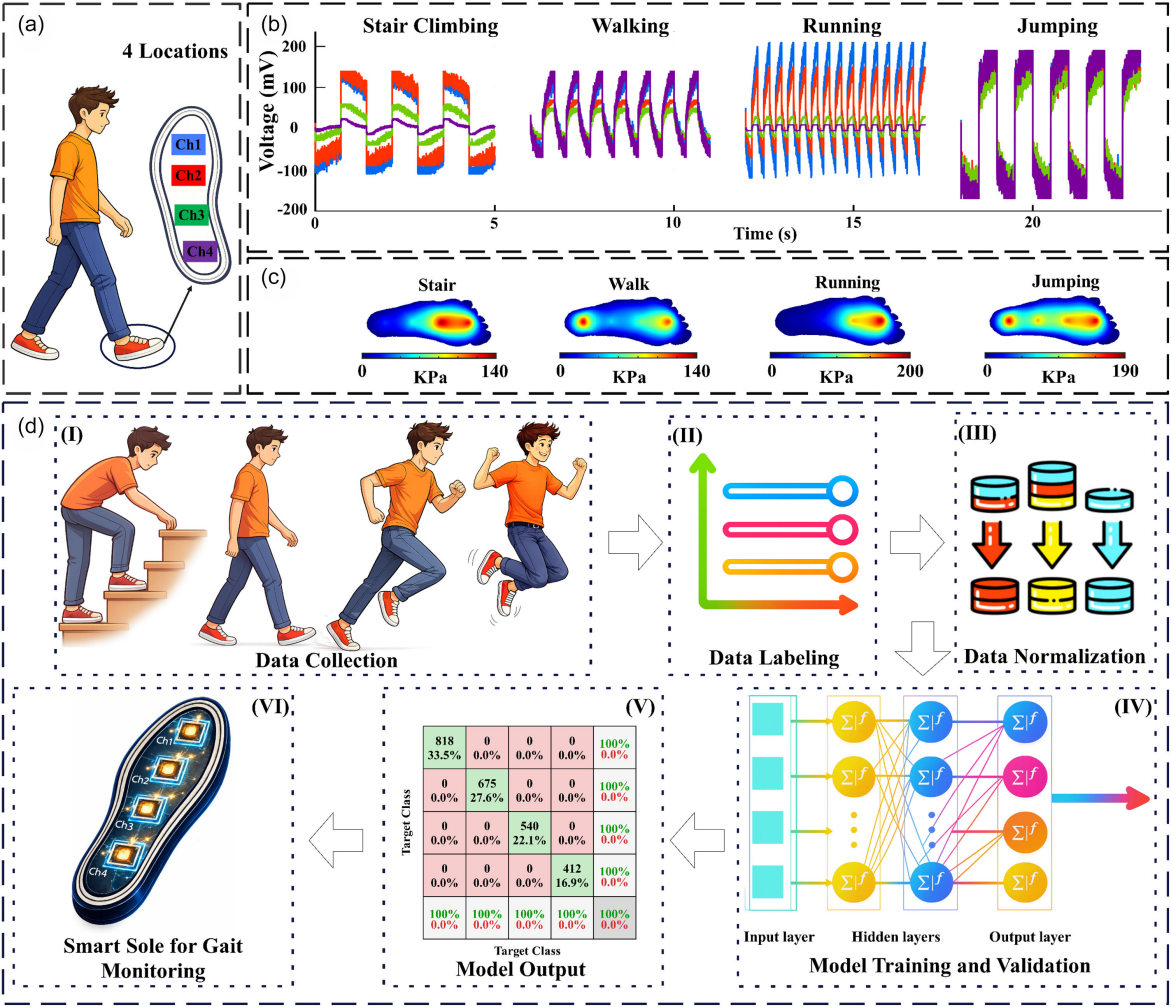
Application demonstration of P3HB:ZnO composite piezoelectric nanofiber for intelligent gait monitoring: (a) schematic illustration of the smart sole with four spatially distributed sensing locations (Ch1‐Ch4), (b) the corresponding multichannel voltage outputs and (c) reconstructed plantar pressure distribution maps recorded during representative locomotion activities, including stair climbing, walking, running, and jumping; (d) end‐to‐end gait recognition workflow, encompassing multiactivity data acquisition using the smart sole, signal labeling and normalization, ANN‐based model training and validation, and classification outputs visualized via a confusion matrix for real‐time gait identification and monitoring.

The potential of the as‐developed biodegradable P3HB:ZnO composite piezoelectric nanofiber for gait monitoring was demonstrated, as a proof‐of‐concept, by integrating it into a smart sole. To evaluate the capability of the system for intelligent gait recognition, an AI‐driven feed‐forward backpropagation neural network was implemented to classify human locomotion patterns. The dataset comprised multichannel voltage signals acquired from four embedded piezoelectric sensing nodes (Ch1‐Ch4) during distinct locomotion activities, including stair climbing, walking, running, and jumping. The corresponding multichannel outputs (Figure 10a) exhibit distinct voltage signatures in terms of amplitude, frequency, and temporal profiles, reflecting differences in foot–ground interaction dynamics (Figure 10b). From these signals, representative temporal and amplitude‐based features were extracted to capture activity‐dependent variations in plantar loading (Figure 10c). To classify data, neural network consisted of an input layer receiving the processed features, followed by two hidden layers with 25 and 15 neurons, respectively, employing hyperbolic tangent sigmoid activation functions utilized to capture nonlinear relationships between biomechanical inputs and locomotion states (Figure 10d). The output layer used a softmax activation function to classify the signals into four activity categories corresponding to the gait modes. Model training was performed using the scaled conjugate gradient backpropagation algorithm [54]. Cross‐entropy was used as the loss function to optimize classification performance and generalization. The machine‐learning model was developed using more than 4000 data points, which were randomly divided into training (85%), validation (5%), and testing (10%) subsets. The trained model achieved excellent classification accuracy (step V, Figure 10d), as demonstrated by the confusion matrix, where values are concentrated along the main diagonal and off‐diagonal entries are nearly zero, indicating that most predictions match the true labels with minimal misclassification.

This confirms robust discrimination among stair climbing, walking, running, and jumping. Nevertheless, it should be mentioned that larger‐scale validation involving a greater number of participants and more diverse locomotion conditions will be required before practical implementation, and this will be the focus of future studies. Furthermore, although the optimized device demonstrated stable piezoelectric performance over 1000 loading cycles, a comprehensive assessment of its long‐term durability and reliability under real‐world operating conditions is still required. Such evaluation is beyond the scope of the present proof‐of‐concept study and will be addressed in future work.

## 4 Conclusion

This work establishes an integrated, artificial intelligence‐assisted design framework for enhancing the piezoelectric performance of biodegradable P3HB‐based nanofibers reinforced with ZnO nanoparticles. By combining RSM‐guided experimental design with ANN modeling and GA optimization, the complex nonlinear relationships among ZnO concentration, applied voltage, drum speed, and electrospinning time were systematically captured and optimized. The framework identified an optimal ZnO content of 4.3 wt.%, representing a balance between interfacial polarization and structural uniformity. Structural and physicochemical characterization confirmed that the optimized P3HB:ZnO composite piezoelectric nanofiber exhibits a uniform nanofiber morphology (average diameter of 1450 nm), high toughness (37.28 MJ/m^3^), enhanced polarization, and slightly increased surface hydrophilicity (124° compared to 132°). Piezoelectric characterization demonstrated a high voltage sensitivity of 0.776 mV/kPa at 1.75 Hz under dynamic loading, along with stable performance over 1000 loading cycles. Energy‐harvesting evaluation further revealed high performance, with the capacitor charging voltage reaching 31.78 mV at 1.75 Hz under 24 kPa and a peak stored power density of 6.7 W/m^2^. Electrical resistance‐dependent analysis identified an optimal power output of 22.46 W/m^2^ at 2 kΩ, confirming effective impedance matching and efficient mechanical‐to‐electrical energy conversion. Biodegradation studies demonstrated progressive structural degradation, with 38% weight loss observed within 6 weeks while retaining piezoelectric functionality, followed by complete disintegration by week 10. Integration of the optimized nanofibers into a multichannel gait monitoring system validated the potential of the developed piezoelectric materials for intelligent physiological signal interpretation through AI‐assisted analysis.

## Author Contributions

**Milad Razbin:** conceptualization, methodology, investigation, data analysis and modeling, visualization, writing original draft, and review and editing. **Kexin Ruan:** ethodology. **Danish Tahir:** review and editing. **Xuan Li:** review and editing. **Qing Li:** review and editing. **Hala Zreiqat:** review and editing. **Fariba Dehghani:** conceptualization, review and editing, funding acquisition, and supervision. **Shuying Wu:** administration, conceptualization, review and editing, funding acquisition, and supervision.

## Funding

This work was supported by the Australian Research Council (DP240101993, IH210100040 and LP230200997), and Faculty of Engineering at University of Sydney.

## Conflicts of Interest

The authors declare no conflicts of interest.

## Data Availability

The data that support the findings of this study are available on request from the corresponding authors.
